# Account of the Sudden Death of an Infant, in Reference to Medical Jurisprudence

**Published:** 1831-06

**Authors:** Joseph Ashley Gaitskell

**Affiliations:** Bath.


### SUDDEN DEATH OF AN INFANT.

*Account of the Sudden Death of an Infant, in reference to Medical Jurisprudence.* By JOSEPH ASHLEY GAITSKELL, M.D., Bath.

MEDICAL jurisprudence having laterly engaged much attention, I shall be gratified if the following case contributes a humble mite in elucidation of that interesting subject.

H—, a very healthy male infant, exactly four months old, was found dead on his bed, at half-past six o'clock of the morning, on the 2d of April, under the following circumstances:

The infant was taken to bed, as usual, with his mother and father, a respectable hairdresser of this city. The mother was in the middle of the bed, the father on one side of her, the infant on the other, placed upon a pillow, and the bed-clothes well tucked in, to prevent him falling out. The party was thus settled at twelve o'clock at night. The father had been out to spend the evening, the mother had waited up for him, and went to bed very tired. The father is a strong, hearty man; the mother a very weakly, delicate woman, and deaf; both of them sober, honest, and industrious. In a small adjoining room, with a door of communication between the two, a servant girl and another child slept. Whatever passes in either room, can be heard almost as distinctly in one as in the other. The servant has lived with the family several years. There was no light in either room.

At half-past six o'clock in the morning, the father and mother awoke, nearly at the same time. The father arose instantly to dress, as was his custom, and the mother looked for her infant: not finding him in his usual place, she exclaimed immediately, "Where's my child?" The father had gone round to the other side of the bed, and hearing the mother's exclamation, looked on the floor, expecting to find it there: meantime the mother had found it, lying midway on the outside of the bed, with its face downwards, its head towards the foot, and the feet towards the head of the bed: it was rather cold, a little patch of red on one side of its face, and its mouth open, as in the act to take the breast. The mother instantly caught up the infant, discovered it was dead, and called quickly to Martha, the servant. The father, not believing it possible the infant could be dead, cried out, "Nonsense!" however, they were soon convinced of the reality, and immediately sent next door for a young gentleman, a hospital pupil. He very properly examined the mouth, which was quite free from obstruction, passed a tube into the air-passages, and endeavoured to restore vitality by inflating the lungs; but without the desired result: he also placed the infant in a warm bath.

At nine o'clock, the father called to tell me what had happened. I desired the body to be opened, which was done by the same young gentleman, in my presence, at twelve o'clock.

Externally: the groins, chest, back about the shoulders, and a very small portion of one cheek, were of that red colour which is often seen in dead bodies. This appearance was perhaps greater in degree, and sooner than usual, in consequence of the infant having been placed in a warm bath before it was quite cold. The young gentleman said, when he first saw it, there were none of these marks.

Internally: on opening the chest, the lungs were so retracted that they could have contained but very little air; their external appearance was perfectly healthy, except a very small portion of the anterior extremity of the middle lobe of the right, which was loaded with dark-coloured blood. The larynx, trachea, and lungs being removed entire, were opened throughout their whole length, and were free from froth or other obstruction, and from every sign of disease. In opening the chest, the superior vena cava was unfortunately largely wounded; consequently, the blood from thence and the right side of the heart was effused into the cavity of the chest. This prevented any observation on the state those parts might have been in; but the blood was exceedingly black, and the quantity such as more than to fill that side of the chest: it seemed as though all the blood in the body must have been concentrated in the right side of the heart and large veins. The left side of the heart was entirely empty. The stomach was tensely distended with wind, but otherwise quite empty; the small intestines were in the same state; the large ones contained some remains of ingesta. All these parts were perfectly healthy in structure, but remarkably pale, neither artery nor vein being visible, either internally or externally. The lacteals were empty. The liver appeared dark-coloured, not from settling of blood in consequence of position, but as though all the veins being filled with black blood, the colour shone through them to the surface, and was diffused equally through every portion of its substance. The kidneys were healthy. The brain was removed entire, and presented the most healthy appearance; the blood within it not darker, nor more in quantity, than usual, but rather the reverse, the cortical part being very pale.

*Observations.* The first question which arises is, how could the infant get into the situation in which it was found. It is certain an infant, only four months old, could not rise from its mother's side, and by any accident place itself there: the whole family had been asleep, and awoke quite unconscious of what had happened. The only explanation the mother can give is, that she, in her sleep, must have taken up the infant, and laid it down outside the bed, quite unconscious of what she was doing: this is the more probable, as she had done a similar thing once or twice before with the same child. I think the infant might have wished to suck, and by its efforts disturbed its mother, who, being over-tired, took it up mechanically, laid it down outside the bed, unfortunately, its face downwards; and then fell asleep again, without the slightest consciousness of what she had done. The infant could not have cried, nor the parents have made any stir, or they would have been overheard by the servant in the adjoining room, who that night had very little rest, in consequence of the child with her not being very well. The servant is quite satisfied there could have been no noise without her hearing it. The parents are honest, sober, industrious people, renting a house at ninety pounds per annum: part they let to lodgers, and themselves have resided in the other part many years. I have known them several years, and am fully satisfied the death was entirely *accidental.* This child was the finest of the family, the mother had better health than usual whilst nursing him; in short, he was her pet child.

Now let us suppose a similar accident to have occurred to a young woman of light character, with an illegitimate child: would there be any signs by which a medical man could lead a jury to a right judgment in deciding whether the death were the result of accident or of design? I apprehend there would not; that a jury would be obliged to derive its conclusion from circumstantial evidence alone, unless the absence of all signs of violence or of disease, both externally and internally, may be considered a sufficient indication of a similar mode of suffocation. It may be supposed an infant, four months old, if it were accidentally placed upon a bed, with its face downwards, would instinctively turn its head to one side, so as to be able to breathe. In this instance, that was not possible, for its face lay turned against its mother's knee, which had, by its weight, formed a cavity in the bed, out of which the infant had not power to extricate itself: probably, its death was both very speedy and without a struggle. If it had been able to cry, it could also have respired. Many infants die by being overlaid: that circumstance would occasion little suspicion of design; but an infant found dead on the outside of the bed might, under certain contingencies, involve doubts of such a nature as to compromise the safety of its unfortunate mother. As the accident has happened where there cannot exist the slightest suspicion of guilt, the remembrance of this case may rèscue an innocent, though perhaps it will contribute little towards convicting a guilty infanticide.

Although it is not the design of this paper to treat of the best method of restoring respiration in cases of asphyxia, yet it may not be out of place to remark, that the inflated state of the stomach and bowels was probably occasioned by misdirected efforts to inflate the lungs, the tube having been passed into the œsophagus, instead of into the larynx.

---

